# Supplementary material for: Computational pan-genomics: status, promises and challenges
Source: Brief Bioinform. 2016 Oct 21;19(1):118–35. doi: 10.1093/bib/bbw089 (PMC5862344; doi:10.1093/bib/bbw089)
Supplement: Supplementary Data [file bbw089_supplement.pdf]

Supplementary Material
Computational Pan-Genomics: Status, Promises and Challenges

List of authors

Tobias Marschall[1,2] Manja Marz[3,4,5,6] Thomas Abeel[7,8] Louis Dijkstra[9,10] Bas E. Dutilh[11,12,13] Ali Ghaffaari[1,2] Paul Kersey[14] Wigard P. Kloosterman[15] Veli Mäkinen[16] Adam M. Novak[17] Benedict Paten[17] David Porubsky[18] Eric Rivals[19,20] Can Alkan[21] Jasmijn A. Baaijens[22] Paul I. W. De Bakker[15] Valentina Boeva[23,24,25,26] Raoul J. P. Bonnal[27] Francesca Chiaromonte[28,29] Rayan Chikhi[30] Francesca D. Ciccarelli[31] Robin Cijvat[32] Erwin Datema[33] Cornelia M. Van Duijn[34] Evan E. Eichler[35,36] Corinna Ernst[37] Eleazar Eskin[38,39] Erik Garrison[40] Mohammed El-Kebir[22,41,42] Gunnar W. Klau[22] Jan O. Korbel[14,43] Eric-Wubbo Lameijer[44] Benjamin Langmead[45] Marcel Martin[46] Paul Medvedev[47,48,29] John C. Mu[49] Pieter Neerincx[44] Klaasjan Ouwens[50,51] Pierre Peterlongo[52] Nadia Pisanti[53,54] Sven Rahmann[37] Ben Raphael[42] Knut Reinert[55] Dick de Ridder[56] Jeroen de Ridder[15] Matthias Schlesner[57] Ole Schulz-Trieglaff[58] Ashley D. Sanders[59] Siavash Sheikhizadeh[56] Carl Shneider[60] Sandra Smit[56] Daniel Valenzuela[16] Jiayin Wang[61,62,63] Lodewyk Wessels[64] Ying Zhang[32,22] Victor Guryev[18] Fabio Vandin[65,66,42] Kai Ye[67,68,63] Alexander Schönhuth[22]

List of affiliations

1 Center for Bioinformatics, Saarland University, Saarland Informatics Campus, 66123 Saarbrücken, Germany
2 Max Planck Institute for Informatics, Saarland Informatics Campus, 66123 Saarbrücken, Germany
3 Bioinformatics and High Throughput Analysis, Faculty of Mathematics and Computer Science, Friedrich Schiller University Jena, Leutragraben 1, 07743 Jena, Germany
4 FLI Leibniz Institute for Age Research, Beutenbergstraße 11, 07745 Jena, Germany
5 Michael Stifel Center Jena, Ernst-Abbe-Platz 2, 07743 Jena, Germany
6 German Centre for Integrative Biodiversity Research (iDiv) Halle-Jena-Leipzig
7 Delft Bioinformatics Lab, Delft University of Technology, Delft, The Netherlands
8 Broad Institute of MIT and Harvard, Cambridge, Massachusetts, USA
9 Computational Science Lab, University of Amsterdam, Amsterdam, 1098XG, The Netherlands
10 Department of High Performance Computing, ITMO University, Saint Petersburg, 197101, Russia
11 Radboud Institute for Molecular Life Sciences, Center for Molecular and Biomolecular Informatics, Radboud University Medical Center, Nijmegen, Netherlands
12 Theoretical Biology and Bioinformatics, Utrecht University, Utrecht, Netherlands
13 Department of Marine Biology, Institute of Biology, Federal University of Rio de Janeiro, Rio de Janeiro, Brazil
14 EMBL-European Bioinformatics Institute, Wellcome Trust Genome Campus, Hinxton, CB10 1SD, UK
15 Department of Genetics, Center for Molecular Medicine, University Medical Center Utrecht, 3584 CG, Utrecht, The Netherlands
16 HIIT and Department of Computer Science, University of Helsinki, Finland
17 UC Santa Cruz Genomics Intsitute, University of California Santa Cruz, Santa Cruz, CA 95064, USA
18 European Research Institute for the Biology of Ageing, University Medical Center Groningen, University of Groningen, Antonius Deusinglaan 1, AV Groningen 9713, The Netherlands
19 LIRMM, CNRS and Université de Montpellier, Montpellier, France
20 Institut de Biologie Computationnelle, CNRS and Université de Montpellier, Montpellier, France
21 Department of Computer Engineering, Bilkent University, Bilkent, Ankara, 06800, Turkey
22 Life Sciences Group, Centrum Wiskunde & Informatica, Amsterdam, 1098XG, The Netherlands
23 Institut Curie, Centre de Recherche, Inserm U900, F-75005 Paris, France
24 Mines ParisTech, F-77305 cedex Fontainebleau, France
25 PSL Research University, F-75005 Paris, France
26 Institut Cochin, Inserm U1016, CNRS UMR 8104, Université Paris Descartes UMR-S1016, F-75014 Paris, France
27 Istituto Nazionale Genetica Molecolare INGM, 'Romeo ed Enrica Invernizzi', Milan 20122, Italy.
28 Department of Statistics, The Pennsylvania State University, University Park, PA, 16802, USA
29 Genome Sciences Institute (Huck Institutes of the Life Sciences), The Pennsylvania State University, University Park, PA, 16802, USA
30 CNRS, Univ. Lille, UMR 9189 CRIStAL, F-59000 Lille, France
31 Division of Cancer Studies, King's College London, London SE11UL, UK

32 MonetDB Solutions, Amsterdam, The Netherlands
33 KeyGene N.V, Agro Business Park 90, 6708 PW Wageningen, The Netherlands
34 Department of Epidemiology, Erasmus Medical Center, Rotterdam, The Netherlands
35 Department of Genome Sciences, University of Washington, Seattle, WA, 98195, USA
36 Howard Hughes Medical Institute, University of Washington, Seattle, WA, 98195, USA
37 Genome Informatics, Institute of Human Genetics, University Hospital Essen, University of Duisburg-Essen, Essen, Germany
38 Department of Computer Science, University of California, Los Angeles, USA
39 Department of Human Genetics, University of California, Los Angeles, USA
40 Wellcome Trust Sanger Institute, Cambridge, UK
41 Centre for Integrative Bioinformatics VU (IBIVU), VU University Amsterdam, De Boelelaan 1081A, 1081 HV Amsterdam, The Netherlands
42 Center for Computational Molecular Biology and Department of Computer Science, Brown University, Providence, RI 02912, USA
43 European Molecular Biology Laboratory (EMBL), Genome Biology Unit, Meyerhofstrasse 1, 69117 Heidelberg, Germany
44 Genomics Coordination Center, University of Groningen, University Medical Center Groningen, Groningen, 9700RB, The Netherlands
45 Department of Computer Science and Center for Computational Biology, Johns Hopkins University, Baltimore, Maryland
46 Science for Life Laboratory, Dept. of Biochemistry and Biophysics, Stockholm University, Box 1031, SE-17121 Solna, Sweden
47 Department of Computer Science and Engineering, The Pennsylvania State University, University Park, PA, 16802, USA
48 Department of Biochemistry and Molecular Biology, The Pennsylvania State University, University Park, PA, 16802, USA
49 Bina Technologies, Roche Sequencing, Redwood City, CA 94065, USA
50 Biological Psychology, Vrije Universiteit Amsterdam, The Netherlands
51 Genalice BV, Harderwijk, The Netherlands
52 IRISA Inria Rennes Bretagne Atlantique, GenScale team, Campus de Beaulieu, 35042 Rennes, France
53 Dipartimento di Informatica, University of Pisa, Pisa, Italy
54 Erable Team, INRIA
55 Department of Mathematics and Computer Science, Freie Universität Berlin, Berlin, Germany
56 Bioinformatics Group, Wageningen University, Droevendaalsesteeg 1, 6708 PB, Wageningen, The Netherlands
57 Division of Theoretical Bioinformatics, German Cancer Research Center (DKFZ), Im Neuenheimer Feld 280, 69120 Heidelberg, Germany
58 Illumina Cambridge Ltd, Chesterford Research Park, Little Chesterford, Essex CB10 1XL, UK
59 Terry Fox Laboratory, BC Cancer Agency, Vancouver, British Columbia, Canada
60 Leiden Observatory, Leiden University, P.O. Box 9513, 2300 RA Leiden, The Netherlands
61 School of Management, Xi'an Jiaotong University, Xi'an, Shaanxi 710049, China
62 Institute of Data Science and Information Quality, Xi'an Jiaotong University
63 Shaanxi Engineering Research Center of Medical and Health Big Data, Xi'an Jiaotong University, Xi'an, Shaanxi, 710049, China
64 Netherlands Cancer Institute (NKI), Amsterdam, the Netherlands
65 Department of Information Engineering, University of Padova, Via Gradenigo 6/B, I-35131, Padova, Italy
66 Department of Mathematics and Computer Science, University of Southern Denmark, Campusvej 55, DK-5230, Odense M, Denmark
67 School of Electronic and Information Engineering, Xi'an Jiaotong University, Xi'an, Shaanxi 710049, China
68 The First Affiliated Hospital of Xi'an Jiaotong University, Xi'an, Shaanxi, 710061, China
